# Determinants of institutional delivery in Sub-Saharan Africa: findings from Demographic and Health Survey (2013–2017) from nine countries

**DOI:** 10.1186/s41182-021-00335-x

**Published:** 2021-05-26

**Authors:** Shewayiref Geremew Gebremichael, Setegn Muche Fenta

**Affiliations:** Department of Statistics, Debre Tabor University, Debre Tabor, Ethiopia

**Keywords:** Institutional Delivery, Sub-Saharan Africa, Women, DHS

## Abstract

**Introduction:**

Institutional delivery is a major concern for a country’s long-term growth. Rapid population development, analphabetism, big families, and a wider range of urban-rural health facilities have had a negative impact on institutional services in Sub-Saharan Africa (SSA) countries. The aim of this study was to look into the factors that influence women’s decision to use an institutional delivery service in SSA.

**Methods:**

The most recent Demographic and Health Survey (DHS), which was conducted in nine countries (Senegal, Ethiopia, Malawi, Rwanda, Tanzania, Zambia, Namibia, Ghana, the Democratic Republic of Congo) was used. The service’s distribution outcome (home delivery or institutional delivery) was used as an outcome predictor. Logistic regression models were used to determine the combination of delivery chances and different covariates.

**Results:**

The odds ratio of the experience of institutional delivery for women living in rural areas vs urban area was 0.44 (95% confidence interval (CI) 0.41–0.48). Primary educated women were 1.98 (95% CI 1.85–2.12) times more likely to deliver in health institutes than non-educated women, and secondary and higher educated women were 3.17 (95% CI 2.88–3.50) times more likely to deliver in health centers with facilities. Women aged 35–49 years were 1.17 (95% CI 1.05–1.29) times more likely than women aged under 24 years to give birth in health centers. The number of ANC visits: women who visited four or more times were 2.98 (95% CI 2.77–3.22) times, while women who visited three or less times were twice (OR = 2.03; 95% CI 1.88–2.18) more likely to deliver in health institutes. Distance from home to health facility were 1.18 (95% CI 1.11–1.25) times; media exposure had 1.28 (95% CI 1.20–1.36) times more likely than non-media-exposed women to delivery in health institutions.

**Conclusions:**

Women over 24, primary education at least, urban residents, fewer children, never married (living alone), higher number of prenatal care visits, higher economic level, have a possibility of mass-media exposure and live with educated husbands are more likely to provide health care in institutions. Additionally, the distance from home to a health facility is not observed widely as a problem in the preference of place of child delivery. Therefore, due attention needs to be given to address the challenges related to narrowing the gap of urban-rural health facilities, educational level of women improvement, increasing the number of health facilities, and create awareness on the advantage of visiting and giving birth in health facilities.

## Introduction

From 2000 to 2017, maternal deaths decreased by 38%, implying an annual decrease of 2.9% [1]. The maternal mortality rate (MMR) in developing countries is roughly 40 times higher than in Europe. In 2017, one in every 37 mothers was at risk of maternal death, accounting for roughly 66% of all maternal deaths worldwide [1, 2]. Sub-Saharan Africa is one sub-region with a very large MMR. With a direct emphasis on reducing maternal mortality, the international community planned and accomplished Sustainable Development Goals (SDGs).

Institutional delivery is a major concern for a country’s long-term growth. As a result, the country’s government is preoccupied with maternal and newborn welfare. To improve mother and child health, adequate access to health care during and after pregnancy is required [3]. Birth rates in qualified health centers increased from 6% in 2000 to 28% in 2016 in Ethiopia [3], from 44% in 2008–2009 to 62% in 2014 in Kenya [4], from 42% in 1988 to 74% in 2014 in Ghana [5] and from 51% in 2010 to 64% in 2015–2016 in Tanzania [6]. Increased institutional distribution is critical for lowering maternal and infant mortality. The use of institutional health care is failing to develop in all of Sub-Saharan Africa’s countries. In suburban areas, access to health care is different. In comparison to urban areas, rural areas were less frequent. The explanation for this was a lack of access, distance, and sufficient equipment [3–6]. The explanation for this was a lack of access, distance, and sufficient equipment.

Fast population growth in Sub-Saharan African countries, a high level of analphabetism, a high birth-related presence, and a higher share of the population living in rural areas, where public health centers are inaccessible, have all had a negative impact on institutional delivery systems. Lower economic development, sluggish investment, decreased productivity, and overweight rural residents continue to afflict the region [7]. Increased health care costs as a result of improved economic growth [8, 9]. Increased health care spending results in less under-five deaths, lower maternal morbidity and mortality, better household health, and less public health complications. Since childbirth is difficult, maternal health is the most important topic among these pick points. Pre-presentation and post-presentation (post-natal) ANC visits, as well as useful details on how to care for herself and the infant, are critical for dealing with any problems that arise during childbirth and providing the mother with. Institutional delivery services are influenced by a variety of factors at the individual and community levels. The rate of non-use of institutional delivery services is highest among women with no schooling, low-income households aged 25 to 34 years, who had never married, had never visited an ANC before, and had lived in economically disadvantaged groups [10].

Previous research looked into the factors that influence where a baby is born. However, the national populations of women living in Sub-Saharan African countries have yet to be presented in depth and illustrated. Previous research has shown differences in childbirth between nations, as a result of administrative or other differences. The study's key research issues include those that have been of great importance to institutional provision for women in Sub-Saharan African countries, as well as the determinants (socio-demographic, economic, maternal or other related factors). We are now very inspired to look into the social-demographic, economic, maternal, and institutional distribution characteristics of Sub-Saharan African women.

## Methods

### Study setting, data source, and study design

We use data from the most recent Demographic and Health Survey (DHS) to collect institutional delivery services data from nine countries: Senegal in 2017, Ethiopia in 2016, Malawi in 2016, Rwanda in 2015, Tanzania in 2016, Zambia in 2014, Namibia in 2013, Ghana in 2014, and the Democratic Republic of Congo in 2014 (Table 1).

The countries were chosen based on data availability and historical significance. Measure DHS gave the authors permission to download and use these data for this report. The DHS survey was a cross-sectional study that used stratified multistage (mostly two-stage) cluster sampling to sample people across the country.

The Population and Housing Census (PHC) sampling frames were used in the Enumeration Areas (PHC). It had been used as a preliminary cluster sampling method. Random samples of households were taken in the second stage of clustering within each cluster. All subpopulations are fairly represented in the survey results.

The DHS data are open to the public and provide information on maternal, infant, and child mortality, as well as socio-demographic, economic, and health-related variables. We obtained the information from the DHS, which included the location of birth for mothers aged 15 to 49 years, as determined by sampled households in each cluster unit.

### Study variables

The woman questionnaire was used to obtain the dependent variable, which is the place of delivery. The data was gathered from qualified women aged 15 to 49 years old, who were asked questions about their socio-demographic and economic backgrounds (age, sex, education, marital status, and income), birth history, health facility, media exposure, antenatal visits, women and their husbands' job status, and other topics. The dependent variable in this study was registered as a dichotomous variable: home delivery (no) and institutional delivery (yes).

The residence of the families residing in; fathers’ and mothers’ educational status; women’s age (in years at the time of the survey); the number of living children inside the family; the existence of mothers’ occupation; the household head; the income index; the number of antenatal care (ANC) visits during the pregnancy; the distance between home and health facilities; women’s marital status (at the time of the survey); and access to mass media were all considered independent variables. The independent variables are used because they are available in the dataset and have been studied previously. The independent variables were categorize to make the study simpler (Table 2).

**Table 1:** Year of survey and number of women in the nine Sub-Saharan Africa using Demographic and Health Surveys 2013–2017

Country	Year of survey	Number of women
Senegal	2017	3494
Ethiopia	2016	3551
Malawi	2016	3548
Rwanda	2015	3513
Tanzania	2016	3387
Zambia	2014	3525
Namibia	2013	2874
Ghana	2014	3353
Democratic Republic of Congo	2014	3542
Total		**30787**

**Fig. 1:**
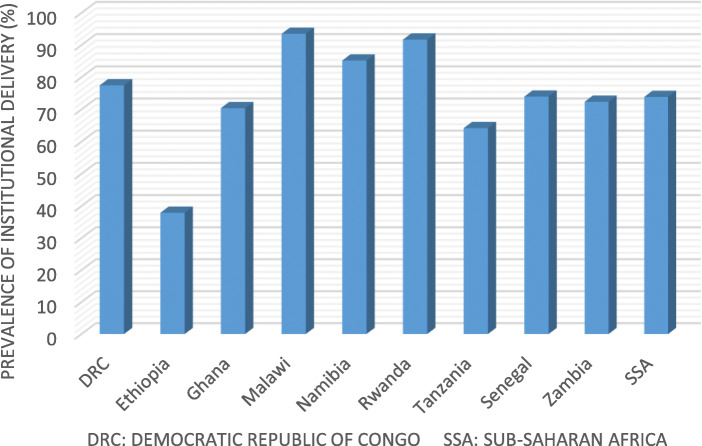
The prevalence of institutional delivery in the nine Sub-Saharan Africa countries from 2013 to 2017. *DRC: Democratic Republic of Congo*

### Statistical analysis

The relation between the odds of the place of delivery and the aforementioned explanatory variables was estimated using Pearson chi-square (X2) and logistic regression models. STATA 14 was used to perform the data analysis. At the univariable point, the chi-square test of association was used to statistically test whether there was a meaningful association between the place of delivery and other explanatory categorical variables or not. As an outcome to the logistic regression model, a binary outcome (home delivery (no) or institutional delivery (yes) is used. The availability of a meaningful effect or correlation of independent variables with the outcome variable is tested using a p-value less than 0.05 or 5%.

### Ethical considerations

The data analyses conducted using the publicly available data of the 2013–2017 DHS of nine Sub-Saharan African countries (https://dhsprogram.com/Data/terms-of-use.cfm). The DHS program has given a written permission letter.

## Results

A total of 30,787 households were included in nine countries. Ethiopia had a national coverage of institutional delivery for women aged 15 to 49, ranging from 37.9 to 93.5%. Malawi had a national coverage of institutional delivery for women aged 15 to 49, ranging from 37.9% to 93.5%. Without taking into account population weighting, the total institutional distribution coverage for all nine countries was 82.3% (Fig. 1).

### Socio-demographic characteristics

We investigated whether there was a significant relationship between institutional delivery and a variety of categorical independent characteristics in the univariable analyses process. Residence, women’s education, age, number of living children, women’s occupation, sex of household head, wealth index, number of ANC visits, husband’s education, distance from home to health facility, marital status, and media exposure with institutional delivery services all showed statistically significant differences (Table 2).

**Table 2:** Relationship between correlates and place of delivery in nine Sub-Saharan Africa

Variable	Place of delivery		Total	*p* value
	Home	Institutional delivery		
	N (%)	N (%)	N (%)	
**Residence**				
Urban	823(10.2)	8252(36.3)	9075(29)	*<* 0.001
Rural	7,211(89.8)	14,501(63.7)	21,712(71)	
**Women education**				
No education	4048(50.4)	4545(20.0)	8593(28)	*<* 0.001
Primary	3113(38.7)	10,187(44.8)	13,300(43)	
Secondary and above	873(10.9)	8021(35.2)	8894(29)	
**Age of women in years**				
15–24	1981(24.7)	6539(28.7)	8520(28)	*<* 0.001
25–34	3840(47.8)	10,986(48.3)	14,826(48)	
35–49	2213(27.5)	5228(23.0)	7441(24)	
**Number of living children**				
0–4	4930(61.4)	17,834(78.4)	22,764(74)	*<* 0.001
Five and above	3104(38.6)	4919(21.6)	8023(26)	
**Women occupation**				
No	3451(43.0)	7722(34.0)	11,173(36)	*<* 0.001
Yes	4583(57.0)	15,031(56.0)	19,614(64)	
**Sex of household head**				
Male	6488(80.8)	17,227(75.7)	23,715(77)	*<* 0.001
Female	1546(19.2)	5526(24.3)	7072(23)	
**Wealth index**				
Poor	5637(70.2)	9233(40.6)	14,870(48)	*<* 0.001
Middle	1377(17.1)	4607(20.2)	5984(19)	
Rich	1020(12.7)	8913(39.2)	9933(32)	
**Number of ANC visit**				
No antenatal visits	2884(36.0)	3193(14.0)	6077(20)	*<* 0.001
1–3	2725(33.9)	7,469(32.8)	10,194(33)	
4 and above	2425(30.1)	12,091(53.2)	14,516(47)	
**Husbands education**				
No education	3644(45.4)	6382(28.0)	10,026(33)	*<* 0.001
Primary	2788(34.7)	7939(35.0)	10,727(35)	
Secondary and above	1602(19.9)	8432(37.0)	10,034(32)	
**Distance to a health facility**				
Big problem	5280(65.7)	12,503(55.0)	17,783(58)	*<* 0.001
Not a big problem	2754(34.3)	10,250(45.0)	13,004(42)	
**Marital status**				
Married	6311(78.6)	15,436(67.8)	21,747(71)	*<* 0.001
Others*	1723(21.4)	7317(32.2)	9040(29)	
**Media exposure**				
Not exposed	4494(56.0)	8251(36.3)	12,745(41)	*<* 0.001
Exposed to mass media	3540(44.0)	14,501(63.7)	18,041(59)	

*Single, widowed, divorced

### Determinants of institutional delivery in Sub-Saharan African countries

In the binary logistic regression model, we examined the possible determinants of institutional services (Table 3). Institutional presence was associated with residency, women’s education, women’s age, number of living children, women’s occupation, household gender, the wealth index, ANC visits, wife education, distance from medical facilities, women’s marital status, media exposure, and national variation among women aged 15 to 49. The odds ratio of the experience of institutional delivery for women living in rural areas vs urban area was 0.44 (95% confidence interval (CI) 0.41–0.48). Primary educated women were 1.98 (95% CI 1.85–2.12) times more likely to deliver in health institutes than non-educated women, and secondary and higher educated women were 3.17 (95% CI 2.88–3.50) times more likely to deliver in health centers with facilities. Women aged 35–49 years were 1.17 (95% CI 1.05–1.29) times more likely than women aged under 24 years to give birth in health centers. The number of ANC visits: women who visited four or more times were 2.98 (95% CI 2.77–3.22) times, while women who visited three or less times were twice (OR = 2.03; 95% CI 1.88–2.18) more likely to deliver in health institutes. Distance from home to health facility were 1.18 (95% CI 1.11–1.25) times; media exposure had 1.28 (95% CI 1.20–1.36) times more likely than non-media-exposed women to delivery in health institutions. The odds of distance from home to health facility indicated that as it was not a big problem to deliver in institutions with health facility (OR = 1.18; 95%CI 1.11–1.25).

**Table 3:** Multivariable logistic regression analysis of factors associated with institutional delivery in Sub-Saharan Africa countries from 2013 to 2017

Variable	Institutional delivery	
	Odds ratio	95% confidence interval
**Residence**		
Urban	1	
Rural	0.44*	0.41–0.48
**Women education**		
No education	1	
Primary	1.98*	1.85–2.12
Secondary and above	3.17*	2.88–3.50
**Age of women in years**		
15–24	1	
25–34	1.01	0.94–1.09
35–49	1.17*	1.05–1.29
**Number of living children**		
0–4	1	
Five and above	0.61*	0.56–0.66
**Women occupation**		
Housewife	1	
Employed	1.34*	1.26–1.43
**Sex of household head**		
Male	1	
Female	1.14*	1.05–1.23
**Wealth index**		
Poor	1	
Middle	1.36*	1.26–1.47
Rich	2.15*	1.97–2.35
**Number of ANC visit**		
No antenatal visits	1	
1–3	2.03*	1.88–2.18
Four and above	2.98*	2.77–3.22
**Husbands education**		
No education	1	
Primary	1.32*	1.23–1.42
Secondary and above	1.22*	1.12–1.32
**Distance to a health facility**		
Big problem	1	
Not a big problem	1.18*	1.11–1.25
**Marital status**		
Married	1	
Others**	1.33*	1.24–1.43
**Media exposure**		
Not exposed	1	
Exposed to mass media	1.28*	1.20–1.36
**Country**		
Democratic Republic of Congo	1	
Ethiopia	0.18*	0.16–0.20
Ghana	0.69*	0.62–0.77
Malawi	4.21*	3.60–4.92
Namibia	1.67*	1.47–1.90
Rwanda	3.23*	2.80–3.73
Tanzania	0.52*	0.47–0.58
Senegal	0.82*	0.74–0.92
Zambia	0.76*	0.68–0.85

*Significant association

**Single, widowed, divorced

Regarding country-level institutional delivery: Ethiopia (82.3%), Ghana (30.9%), Tanzania (47.9%), Senegal (17.6%), and Zambia (23.7%) were less likely to deliver in health institutions than the Democratic Republic of Congo. However, Malawi 4.21 times (OR = 4.21), Namibia 1.7 times (OR = 1.67), and Rwanda 3.23 times (OR = 3.23) more likely delivered in health institutions than the Democratic Republic of Congo (as a reference).

## Discussions

Analyzing the publicly available DHS dataset from nine Sub-Saharan African countries, we have found a nationwide variation of institutional delivery coverage among women aged 15 to 49 years. The institutional delivery can be affected by different socio-demographic and economic characteristics of households. Ethiopia had the lowest use of institutional provision (37.9%), around 82.3% lower than the Democratic Republic of Congo. Malawi, however, has the best institutional results, with 4.2 times more than the Democratic Republic of Congo. In Ethiopia and Malawi, the usage of institutional facilities was poor relative to most Sub-Saharan African countries [11]. Non-users of medical facilities made the most significant contribution during pregnancy and childbirth, as well as highest maternal mortality rate was observed in Malawi [12]. Even if increased institutional delivery reduces maternal and neonatal mortality rates, distance, inaccessibility, and a lack of appropriate facilities make it more difficult [3]. It is worse in rural areas than in urban areas. In most nations, trends have resulted in an increase in the delivery of health services. We found that households with higher educational levels (women and husbands) are more likely to provide institutional care. Previous research has found similar determinants of institutional delivery [12–19]. Parents’ educational qualifications and the survival status of their family members are also associated factors. Earlier research has shown that trained mothers are more knowledgeable of child health and hygiene than their non-educated peers. In Sub-Saharan African countries, the death rate for children under the age of five has decreased [20], Malawi and Uganda [21], Madagascar [22], Tanzania [23], and Nigeria [24].

We have investigated that women’s age has an effect on institutional provision. The older the woman (35 to 49 years old), the more likely she is to give birth in a hospital. The findings were also consistent with previous research [25] and were linked to only mothers under the age of 40, as well as the youngest (15–19 years) and older (40–49 years) mothers. It is difficult to draw broad conclusions based on the age of women and the success of institutions. Women’s age, on the other hand, has been related to institutional service delivery in studies. The health care facility’s service was deteriorating as women grew older [26–29].

The findings revealed that the more live children in a household, the lower the institutional delivery of mothers was statistically important. This is because having a bigger family may have a negative impact on the household’s economic situation. In health facilities, working women are administered more often than women who are not employed.

Women’s households have greater health care practices than men’s households, according to the findings. It may imply the possibility that males can save their own lives and those of their families. The findings also revealed a statistical connection between the wealth index’s status and institutional delivery. The higher a family’s income is, the higher a woman’s income is. Their husbands or other members of their household made decisions based on a lack of financial resources for women, especially in rural areas, which could have an effect on their provision [30–32]. As a result, we might conclude that earnings had the ability to influence where mothers would give birth.

The results revealed that ANC visits are statistically significantly associated with institutional implementation. Women who frequented health facilities prior to delivery may have had an effect on health care. Women who have more ANC visits during their pregnancy are more likely to have ANC visits to health institutions than women who do not have ANC visits [33], at least once [17].

The findings revealed that distance to health care facilities was not a problem. According to our research, the number of single (unpaired) women is higher than the number of married women (paired). The media plays a critical role in the community’s social, economic, and political issues. During our investigation, women from media-exposed groups were found to be significantly associated with institutional growth. Different institutions performed differently across countries.

## Conclusion

The United Nations (UN) [34] published on a nation’s general health and well-being as a proxy for children’s and mothers’ health in 2010. Maternal health and child survival are two sides of the same coin; one cannot exist without the other. Mothers’ and children’s mortality and morbidity can be minimized if proper treatment is taken during pregnancy, childbirth, and postnatal care.

Countries planned and collected data on the assessment of female data at each level of the DHS program, including how women receive treatment (during pregnancy, at work, during childbirth, and in the postnatal period) by asking questions about prenatal, labor, and postnatal delivery of women aged 15 to 49 who had given birth in the previous 5 years.

When women become sick, a variety of factors may make it difficult for them to seek medical help. The knowledge on such determinant factors is particularly important in order to recognize and resolve the inequalities that women may face in obtaining treatment prior to childbirth, during pregnancy, and after birth. The DHS survey of 9 African Sub-Saharan women aged 15 to 49 to see if any of the following variables presented a significant problem during childbirth: distance from the health care center, pre-presentation visits to the health center (ANC), and media exposure.

Women who gave birth in health care facilities accounted for 74% of the total, while the rest (26%) gave birth in the absence of such facilities. In terms of country-level comparisons, Ethiopia has the smallest institutional delivery (37.1%) and Malawi has the largest institutional delivery (93.5%). Women: over 24, at least elementary school, urban, less children; never married (live alone); higher prenatal care visits; increased economic status; had a chance to attend the mass media; had more chances than others to give birth to literary husbands in health-care institutions. Working women were more likely than unemployed women to give birth to their babies with good health facility. A big problem was listed by approximately 58% of respondents; the remaining (42%) did not mention a big problem in the choice of the place of delivery of childhood.

The sub-regional member states should make additional efforts to meet the national and international targets set out in the Sustainable Development Goals (SDG) for reducing maternal mortality by 2030. As a result, the challenges of closing the gap between urban and rural health care, improving women’s educational levels, expanding the number of health care institutions, and raising awareness of visiting and giving birth in health care facilities must all be tackled.

## Data Availability

In accordance with IRB-approved DHS public-use data settings, the data sets analyzed during the current study are available and did not enable respondents, families, or sample communities to be identified in any way. The data files do not contain names of people or household addresses. Only regional levels can be achieved by geographical identifiers (where regions are typically very large geographical areas covering several countries/provinces). There is a PSU number in the data file in each enumeration area (primary sampling unit), but there are no PSU numbers which indicate names or locations. The coordinates are for the entire enumeration area, and not individual households, only in surveys that collect GIS in the field, and the measured coordinates are randomly dispatched within a large geo-area, thus preventing the identification of particular enumeration areas.

## Abbreviations

ANC: Antenatal care
DHS: Demographic and Health Survey
MMR: Maternal mortality rate
OR: Odds ratio
PHC: Population and Housing Census
SDG: Sustainable Development Goals
